# Supplementary material for: Complex‐centric proteome profiling by SEC‐SWATH‐MS
Source: Mol Syst Biol. 2019 Jan 14;15(1):e8438. doi: 10.15252/msb.20188438 (PMC6346213; doi:10.15252/msb.20188438)
Supplement: Supplementary file 8 — Dataset EV7 [file MSB-15-e8438-s008.zip › feature_plots_string/Q9NTJ3.pdf]

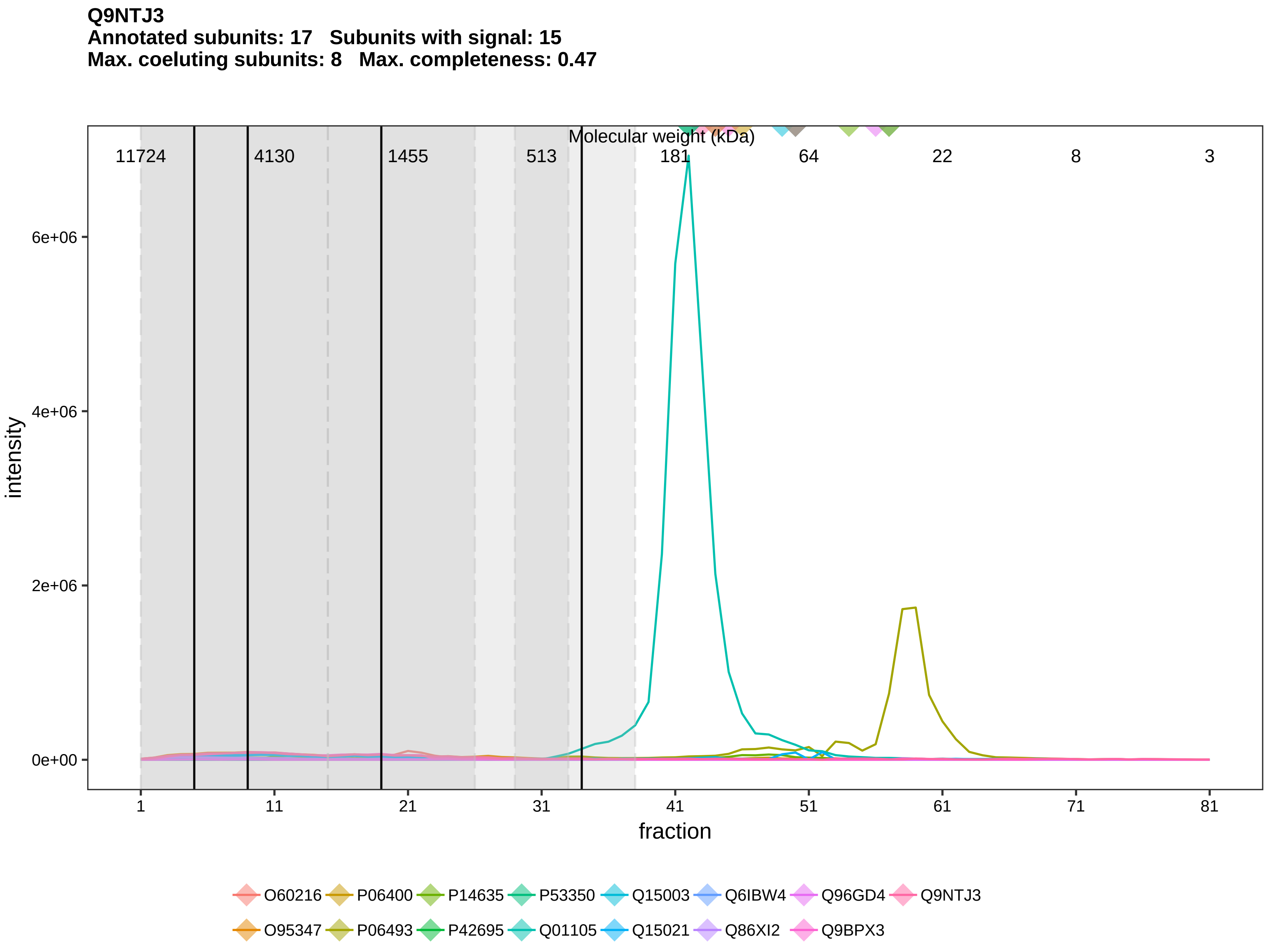
Q9NTJ3
Annotated subunits: 17 Subunits with signal: 15
Max. coeluting subunits: 8 Max. completeness: 0.47
Molecular weight (kDa)
11724
4130
1455
513
181
64
22
8
3
6e+06
4e+06
2e+06
0e+00
intensity
1
11
21
31
41
51
61
71
81
fraction
O60216
P06400
P14635
P53350
Q15003
Q6IBW4
Q96GD4
Q9NTJ3
O95347
P06493
P42695
Q01105
Q15021
Q86XI2
Q9BPX3
